# Ultrasensitive Detection of Ferulic Acid Using Poly(diallyldimethylammonium chloride) Functionalized Graphene-Based Electrochemical Sensor

**DOI:** 10.1155/2014/424790

**Published:** 2014-05-12

**Authors:** Lin-jie Liu, Xia Gao, Pei Zhang, Shi-lan Feng, Fang-di Hu, Ying-dong Li, Chun-ming Wang

**Affiliations:** ^1^School of Pharmacy, Lanzhou University, Lanzhou 730000, China; ^2^Gansu College of Traditional Chinese Medicine, Lanzhou 730000, China; ^3^Department of Chemistry, Lanzhou University, Lanzhou 730000, China

## Abstract

The electrochemical redox of ferulic acid (FA) was investigated systematically by cyclic voltammetry (CV) with a poly(diallyldimethylammonium chloride) functionalized graphene-modified glassy carbon electrode (PDDA-G/GCE) as a working electrode. A simple and sensitive differential pulse voltammetry (DPV) technique was proposed for the direct quantitative determination of FA in *Angelica sinensis* and spiked human urine samples for the first time. The dependence of the intensities of currents and potentials on nature of the supporting electrolyte, pH, scan rate, and concentration was investigated. Under optimal conditions, the proposed sensor exhibited excellent electrochemical sensitivity to FA, and the oxidation peak current was proportional to FA concentration in the range of 8.95 × 10^−8^ M ~5.29 × 10^−5^ M, with a relatively low detection limit of 4.42 × 10^−8^ M. This fabricated sensor also displayed acceptable reproducibility, long-term stability, and high selectivity with negligible interferences from common interfering species. Besides, it was applied to detect FA in *Angelica sinensis* and biological samples with satisfactory results, making it a potential alternative tool for the quantitative detection of FA in pharmaceutical analysis.

## 1. Introduction 

Ferulic acid (FA, 3-methoxy-4-hydroxy cinnamic acid), a ubiquitous bioactive phenolic component in plant tissues, especially in several Chinese medicinal herbs, such as* Angelica sinensis*,* Semen ziziphi spinosae, Cimicifuga heracleifolia*, and* Lignsticum chuangxiong* [1], has been shown to act as a scavenger of various oxidizing species, that is, superoxide anions, hydroxyl radicals, and peroxyl radicals. In view of these biological effects, its several pharmacological activities, such as antioxidant [2], antiaging [3], anti-inflammatory [4], and antithrombotic properties [5], have been widely exploited. In addition, sodium ferulate, a salt of FA, has been used for the treatment of cardiovascular and cerebrovascular diseases [6–8]. In recent years, investigations have focused on the benefits of FA in various fields, including medicine, pharmacy, nutrition, and cosmetics. Therefore, it is of significant importance to develop a fast, simple, effective, and low-cost method for the determination of FA.

Several analytical techniques, including high-performance liquid chromatography [9–11], high-performance capillary electrophoresis [12], and thin-layer chromatography [13], have been used for the quantification of FA. These instrumental methods are not practical for screening large numbers of samples, for they are costly and time-consuming and need complicated equipment. Recently, electrochemically analytical technique has become an alternative method for the detection of FA in the field of pharmaceutical analysis because of its high selectivity, low cost, possible miniaturization, and automation compared with other analytical techniques. Electrochemical behavior and quantitative analysis of FA have been evaluated using several working electrodes, such as glassy carbon electrode (GCE) [14], L-cysteine self-assembled modified gold electrode [15], poly-aspartic acid modified electrode [16], and carbon paste electrode [17]. Nevertheless, it remains a challenge to fabricate an electrochemical sensor based on some novel materials for achieving the sensitive, fast, and facile detection of FA.

In recent years, carbon materials have attracted extensive attention in various fields [18–20]. Graphene, a single layer of carbon atoms in a closely packed honeycomb two-dimensional lattice, is a new kind of carbon nanomaterials with some novel properties such as thermal and chemical stability, superior biocompatibility, high carrier mobility, and outstanding electrical conductivity [21–24]. This unique nanostructure holds great promise for potential applications of graphene in hybrid [25] lithium ion batteries [26, 27] and sensors [28]. In recent years, graphene-based electrochemical sensors have attracted tremendous attention [29–31]. In addition, Yanli Zhang has achieved a rapid electrochemical detection of FA based on a graphene modified glass carbon electrode [32]. However, graphene is prone to agglomerate due to its large specific surface area, which hinders the application of graphene in certain fields. Until now, covalent and noncovalent strategies have been used for the functionalization of graphene to overcome the defect [33, 34], of which noncovalent strategies, particularly using polymers as functional agents, are more favorable than the covalent ones. Liu et al. [35] have gained a stable poly(diallyldimethylammonium chloride) (PDDA) functionalized aqueous dispersion of graphene with the PDDA as stabilizer and hydrazine hydrate as a reducing agent. The solubility of PDDA functionalized graphene (PDDA-G) has greatly increased, which extends the application of graphene in the fields of electrochemical sensors and biosensors. An et al. [36] have fabricated a PDDA-G films modified glassy carbon electrode and achieved sensitive detection of shikonin. As far as we know, there is no report on the application of the PDDA-G based electrochemical sensors for the determination of FA.

In the present work, a simple electrochemical sensor based on PDDA-G modified glassy carbon electrode (PDDA-G/GCE) was fabricated, through which the sensitive determination of FA was achieved. The analytical procedure was based on the electrochemical redox of FA on the electrochemical sensor. This sensor showed excellent electrochemical sensitivity to FA in the selected potential region, and it was applied to detection of FA in* Angelica sinensis* and urine samples containing a certified amount of FA with good selectivity and acceptable reproducibility. The simplicity of this device suggested that it may be a candidate for the rapid screening of FA in real samples, which would extend the application of graphene-based nanocomposite in the fields of pharmaceutical analysis and biosensors.

## 2. Experimental

### 2.1. Materials and Apparatus

Graphite powder (KS-10, 99.5%) and poly(diallyldimethylammonium chloride) (PDDA, *Mw* = 200000 – 350000) were from Sigma; FA was purchased from the National Institute of China for the Control of Pharmaceutical and Biological Products: the stock solution was prepared in methanol, stored in the dark at 4°C, and diluted to the required concentration with the acetate buffer at the time of experiments; hydrazine hydrate (98%), K_2_S_2_O_8_ (99%), P_2_O_5_ (99%), H_2_O_2_ (30%), and concentrated sulfuric acid (A.R. grade) were from Tianjin Guangfu Fine Chemical Industry Research Institute (Tianjin, China). All chemical reagents were of analytical grade, and all solutions were prepared with ultrapure water. 0.1 M HAc and 0.1 M NaAc were used to prepare acetate buffer. All experiments were processed at room temperature (20°C).

All electrochemical experiments were performed on a CHI 1220A electrochemical workstation (Shanghai CH Instrument, China) with a conventional three-electrode cell. A bare or modified glassy carbon electrode (GCE, *d* = 3 mm) was used as working electrode; a saturated calomel electrode (SCE) and a platinum wire electrode were used as reference electrode and auxiliary electrode, respectively. The pH measurement was performed via a pHs-3B digital pH meter (Shanghai Precision Scientific Instrument, China), which was calibrated with standard buffer solution every day. Z36HK centrifuge (Hermle Labortechnik GmbH, Germany) was used for the centrifugation. Infrared spectra were obtained on a Nicolet NEXUS 670 Fourier transform infrared spectrometer (FTIR). Raman spectra were recorded on a Renishaw Raman microscope (Britain). All electrochemical experiments were performed in solutions deaerated by bubbling pure nitrogen for 10 min before the experiment, and a nitrogen atmosphere was kept over the solution during measurements.

### 2.2. Synthesis of Graphene Oxide

Graphene oxide (GO) was prepared from graphite powder according to a modified Hummers and Offeman method [37, 38]. Approximately, 1 g of graphite powder was mixed with 2.5 g of P_2_O_5_, 2.5 g of K_2_S_2_O_8_, and 12 mL of H_2_SO_4_ for 30 min with continuous stirring. The blend was gradually heated to 80°C for 6 h. After the reaction, the products were cleaned by filtration using ultrapure water to produce preoxidized graphite, which was subjected to reoxidation with concentrated H_2_SO_4_ and KMnO_4_. After reoxidation, 360 mL of redistilled water was added to the reaction system, and the reaction was terminated with 30% H_2_O_2_ (20 mL). The resulting mixture was washed by repeated centrifugation and filtration, initially with 5% HCl aqueous solution and then with distilled water. GO product was obtained after the sample was oven-dried at 60°C for 48 h.

### 2.3. Synthesis of PDDA-G

The obtained GO was dispersed in water to yield a yellow-brown dispersion with the aid of ultrasonication for 2 h, followed by centrifugation to remove any unexfoliated GO. Subsequently, the homogeneous GO dispersion (0.5 mg mL^−1^, 20 mL) was mixed with 0.2 mL of PDDA (20%) solution and was stirred for 30 min. The resulting mixture was further treated with 0.1 mL of hydrazine hydrate (80%) and allowed to react for 24 h at 90°C. Finally, the black PDDA-G sheets were collected by centrifugation and further washed with water.

### 2.4. Fabrication of PDDA-G/GCE

Before the sample was modified, GCE was mechanically polished to obtain a mirror-like finish with 0.3 and 0.05 *μ*m of alumina slurry. The polished GCE was then rinsed thoroughly with doubled-distilled water. GCE was successively sonicated in acetone and double-distilled water to remove residual alumina loosely bound to the electrode surface. This surface was then dried by nitrogen. PDDA-G/GCE was fabricated according to the reported procedure [35, 36].

### 2.5. Electrochemical Measurements

Measurements were performed in an electrochemical cell containing 10 mL of 0.1 M acetate buffer (pH 3.0). For cyclic voltammetry (CV), the sweep was from 0 V to 0.7 V at the scan rate of 100 mV s^−1^. Differential pulse voltammetric (DPV) experiments were carried out from 0 V to 0.7 V with an amplitude of 0.05 V, a pulse width of 0.05 s, an increment potential of 0.004 V, a sample width of 0.01667 s, a pulse period of 0.2 s, and a quiet time of 2 s. The electrode was modified again after each scanning. All calibration curves of FA were performed with a successive standard addition method; calibration plots represented the overall current variation as a function of the substrate concentration.

### 2.6. Sample Treatment

Dried powder of* A. sinensis* (20 g) was accurately weighed and transferred into a 250 mL round-bottom flask. 70% ethanol (160 mL) was added and reflux-extracted for 1.5 h. After filtration, the residue was reflux-extracted again according to the above conditions. The filtrate of amalgamation was concentrated to 50 mL. The concentrated solution (100 *μ*L) was added to the acetate buffer solution of pH 3.0 (10 mL in total), and FA was determined by using DPV with standard addition method.

Urine samples were stored in a refrigerator immediately after collection. The urine samples were 100 times diluted with acetate buffer before analysis without any pretreatments. The recoveries were measured by spiking drug-free urine with known amounts of FA, and the calibration graph was used to determine FA spiked in samples.

## 3. Results and Discussion

### 3.1. Characterizations of PDDA-G

**Figure 1:**
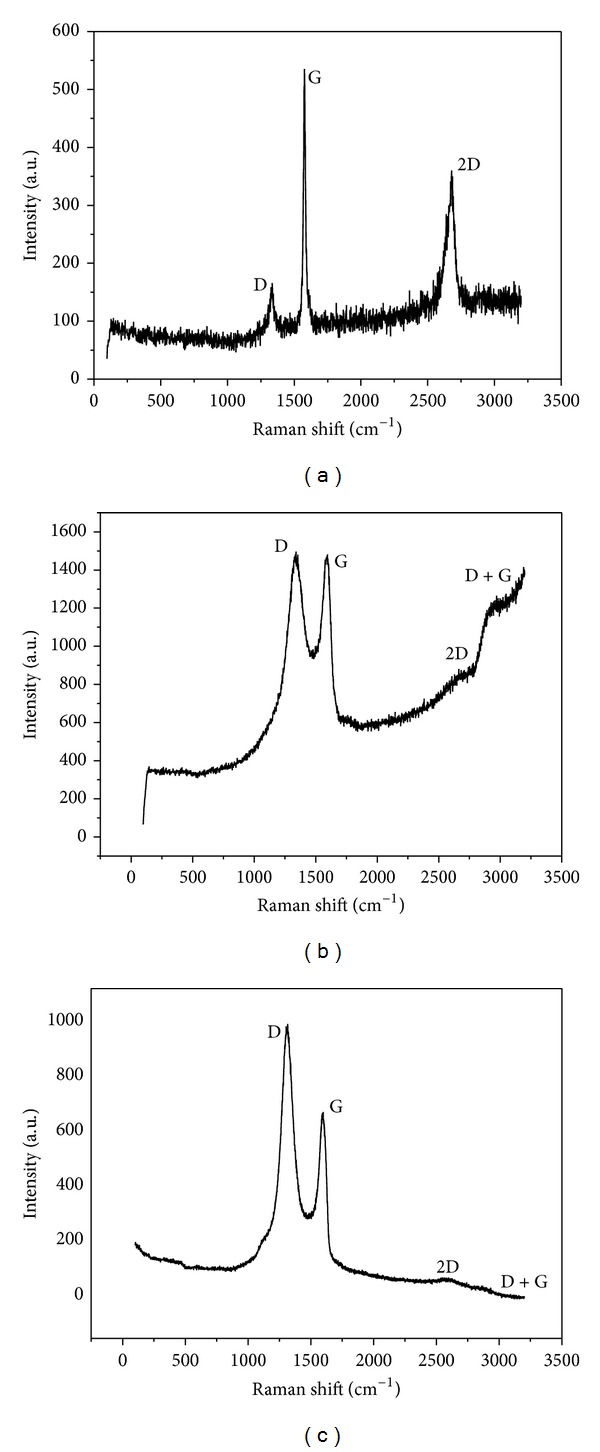
Raman spectra of graphite (a), GO (b), and PDDA-G (c).

Raman spectroscopy was utilized to characterize the morphology and composition of PDDA-G. As shown in Figure 1, the Raman spectra of GO (b) and PDDA-G (c) exhibited a new D+G combination band arising from the cooperation between D and G peaks compared with the natural graphite (a), which indicated the presence of highly disordered and randomly arranged graphene sheets. In addition, the D/G intensity ratio of PDDA-G increased obviously compared with GO and graphite, indicating the restored ordered crystal structure of PDDA-G and the smaller *sp* ^2^ domains in PDDA-G sheets [39]. Moreover, the change in shape of the high energy second-order 2D band at 2674 cm^−1^ indicates the single layer structure and the increase in thickness of PDDA-G, which should be attributed to the attachment of PDDA.

**Figure 2:**
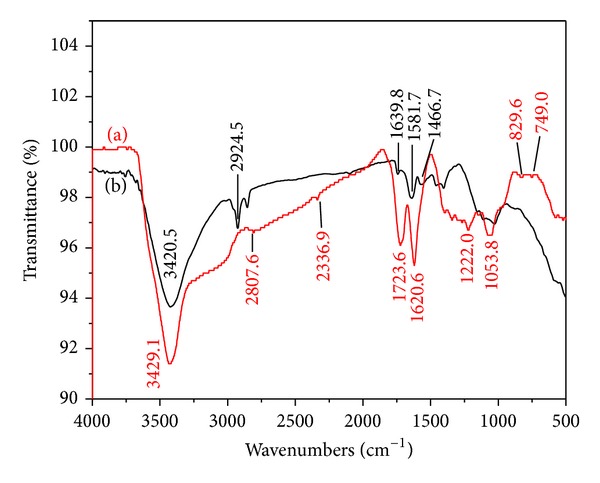
FTIR spectra of GO (a) and PDDA-G (b).

Furthermore, FTIR spectroscopy was also employed to investigate the reduction of the oxygen-containing groups in GO during the functionalized process [36]. As shown in Figure 2, after reduction of the GO (curve a) with hydrazine for 24 h, the FTIR peaks intensities in PDDA-G (curve b) corresponding to the oxygen functionalities, such as the C = O stretching vibration peak at 1724 cm^−1^, the C–O (epoxy) stretching vibration peak at 1222 cm^−1^, the vibration and deformation peaks of O–H groups at 3429 and 1621 cm^−1^, respectively, and the C–O (alkoxy) stretching peak at 1054 cm^−1^, decreased dramatically, and some of them disappeared entirely. These changes indicate that most of the oxygen-containing functional groups of GO were removed during the reduction process with hydrazine. Moreover, the PDDA-G showed an obvious skeletal vibration adsorption band of the graphene at 1582 cm^−1^, and these bands at 2924 (CH_*n*_), 1640 (C = C), and 1467 cm^−1^ (C = C) belong to the characteristic bands of PDDA, indicating the functionalization of graphene with PDDA.

### 3.2. Electrochemical Response of FA to Different Electrodes

**Figure 3:**
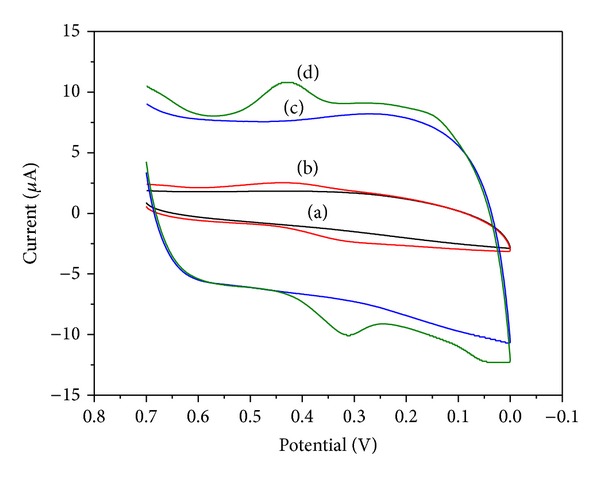
CVs of GCE (a, b) and PDDA-G/GCE (c, d) in acetate buffer solution (0.1 mol L^−1^, pH 3.0) with 5.86 × 10^−6^ M FA (b, d) and without FA (a, c).

Figure 3 shows cyclic voltammograms (CVs) of FA on GCE and PDDA-G/GCE at scan rate of 100 mV** **s^−1^. No redox peak was observed at GCE (a) in the solution without FA. The same phenomenon was also observed in PDDA-G/GCE (c), indicating the electroinactivity of PDDA-G in the selected potential region. After a specific amount of FA was added to the buffer solution, FA showed relatively poor redox peaks in GCE (b), while the redox peaks increased considerably in PDDA-G/GCE at *E*_pa_ = 0.429** **V and *E*_pc_ = 0.311** **V (d). The ratio of the anodic peak current (*I*_pa_) and the cathodic peak current (*I*_pc_) was approximate to 1, revealing a fast electron-transfer process of FA at PDDA-G/GCE. Moreover, there was a large background current at the PDDA-G/GCE when no FA was added, which was ascribed to the larger specific surface area of PDDA-G. These results indicated that the PDDA-G could provide a large specific surface area to increase the loading amount of FA and could accelerate the electron transfer on the electrode surface to amplify the electrochemical signal as a result of its outstanding electric conductivity.

### 3.3. Effect of pH

**Figure 4:**
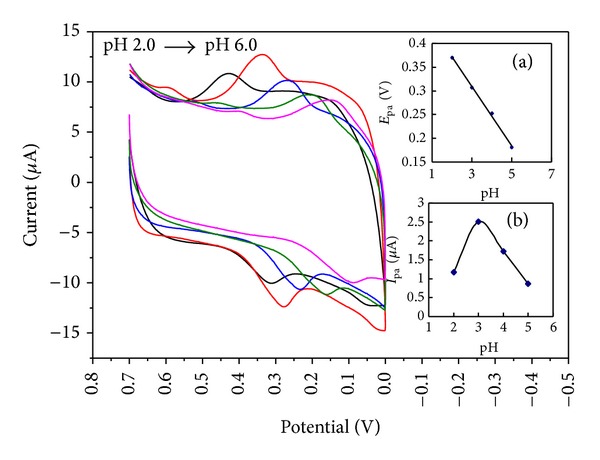
CVs of PDDA-G/GCE in the presence of FA (5.86 × 10^−6^ M) at different pH (2.0, 3.0, 4.0, 5.0, and 6.0); inset: (a) the relationship between the anodic peak potentials and the pH; (b) the relationship between the anodic peak currents and the pH.

The influence of buffer pH on the cyclic voltammetric responses of FA was investigated in pH range from 2.0 to 6.0. As shown in Figure 4, the redox peaks shifted negatively as pH increased. This result indicated an electrochemical process accompanied by protons. A good linear relationship between *E*_pa_ and pH was constructed (a) with a linear regression equation expressed as *E*_pa_ = 0.4955–0.0623 pH, *R* ^2^ = 0.9969 (*E*_pa_: V). According to Nernst equation *E*_pa_= *E* ^*θ*^− [(2.303 mRT)/(nF)] pH [38, 40], the ratio of *m*/*n* was calculated as 1.06; this finding showed that the numbers of transferred electrons and protons were equal during the electrode reaction, and the result was consistent with those in previous reports [14, 16]. Moreover, the maximum oxidation peak current was achieved at pH 3.0 (b). Thus, pH 3.0 was chosen for subsequent analytical experiments.

### 3.4. Effect of Scan Rate

**Figure 5:**
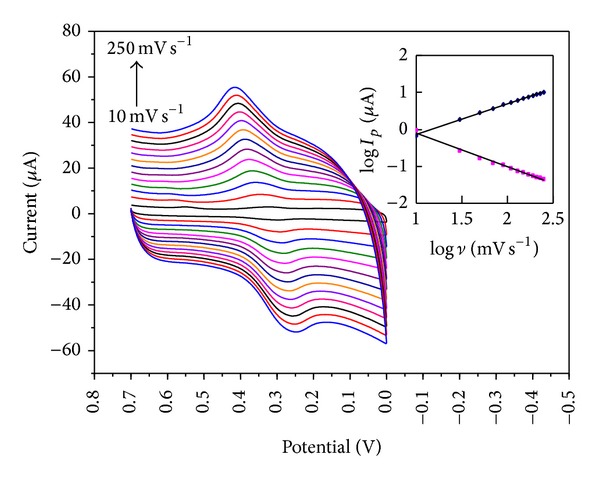
CVs of PDDA-G/GCE in the presence of FA (5.86 × 10^−6^ M) with different scan rates (10, 30, 50, 70, 90, 110, 130, 150, 170, 190, 210, 230, and 250 mV s^−1^) in pH 3.0 acetate buffer solution; inset: the relationship between the logarithm of the peak currents and the logarithm of the scan rates.

**Figure 6:**
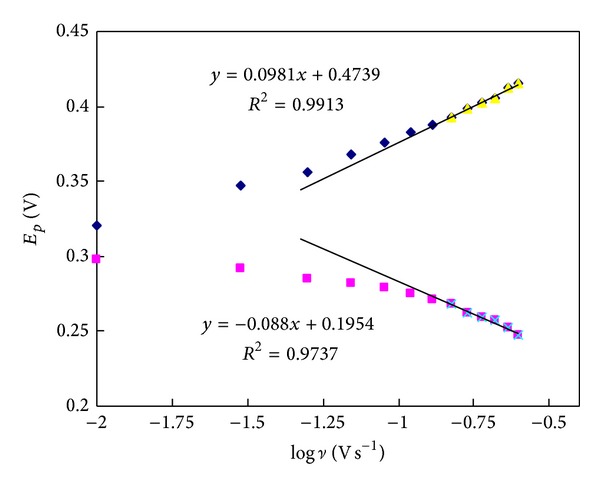
The plots of the anodic and cathodic potentials against the logarithm of scan rates.

The CVs of FA on PDDA-G/GCE at different scan rates were exhibited (Figure 5). The peak currents increased with the increase of scan rates, and a linear dependence was established (inset) between the logarithm of the anodic peak currents and that of the scan rates from 10 mV s^−1^ to 250 mV s^−1^, indicating an adsorption-diffusion mixed controlled electrode process. At higher scan rate, the redox potential had a linear relationship with the logarithm of scan rate (Figure 6), and the regression equations were *E*_pa_ = 0.4739 + 0.0981 log *ν* (*E*_pa_: V, *ν*: V s^−1^) and *E*_pc_ = 0.1954 − 0.088 log *ν* (*E*_pc_: V, *ν*: V s^−1^) with *R* ^2^ = 0.9913 and 0.9737, respectively.

According to Laviron's equations [41],

(1) Epa=Eθ′+2.303RT(1−α)nFlog⁡ ν,Epc=Eθ′−2.303RTαnFlog⁡ ν,log⁡ks=α log⁡(1−α) +(1−α)log⁡α−log⁡RTnFν−α(1−α)nFΔEp2.3RT.

**Scheme 1:**
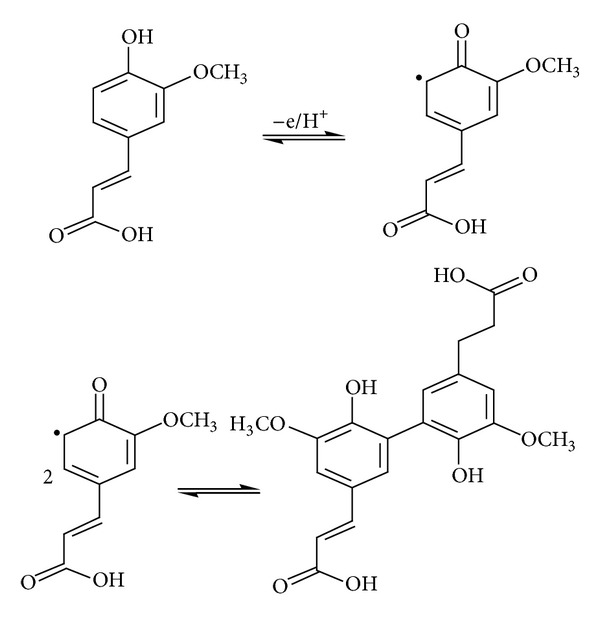
Electrode oxidation reaction equation of FA at electrode surface.

Therefore, the electron-transfer coefficient (*α*), electron-transfer number (*n*), and electrode reaction rate constant (*k*_*s*_) were calculated as 0.53, 1.25, and 0.57 s^−1^, respectively. This result indicated a fast electron-transfer process of FA at PDDA-G/GCE, and a possible reaction mechanism of FA in PDDA-G/GCE is shown in Scheme 1.

### 3.5. Effect of Accumulation Conditions

**Figure 7:**
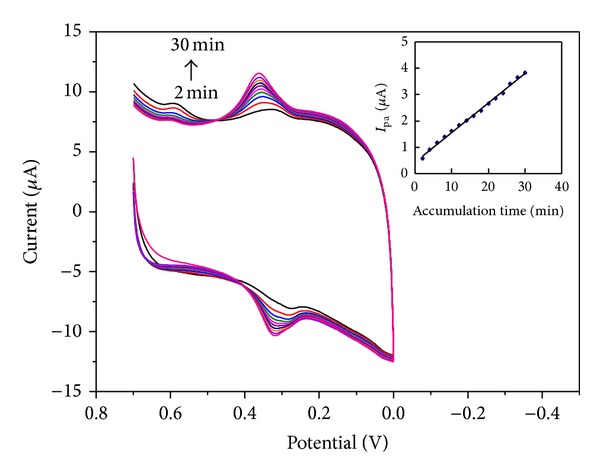
CVs of PDDA-G/GCE in the presence of FA (5.86 × 10^−6^ M) at scan rate of 100 mV s^−1^ with accumulation time varying from 2 min to 30 min in pH 3.0 acetate buffer solution. Inset: the relationship between the anodic peak currents and the accumulation time.

Accumulation can improve the amount of FA absorbed on the electrode surface and thus improve determination sensitivity and decrease detection limit. To evaluate the influence of accumulation time on the determination of FA, we obtained the CVs of FA at PDDA-G/GCE under different accumulation times. As shown in Figure 7, the oxidation peak current increased gradually with the extension of accumulation time from 2 to 30 min. This phenomenon may indicate a polymerization reaction of FA at the electrode surface. Accumulation potential slightly affected the peak current of FA at −0.5 V to 0.5 V, and the potential at approximately 0 V had a slight advantage for obtaining the maximal peak currents. Considering both sensitivity and work efficiency, the accumulation time of 5 min and the accumulation potential of 0 V were employed in further experiments.

### 3.6. Effect of the Immobilized Amount of PDDA-G

**Figure 8:**
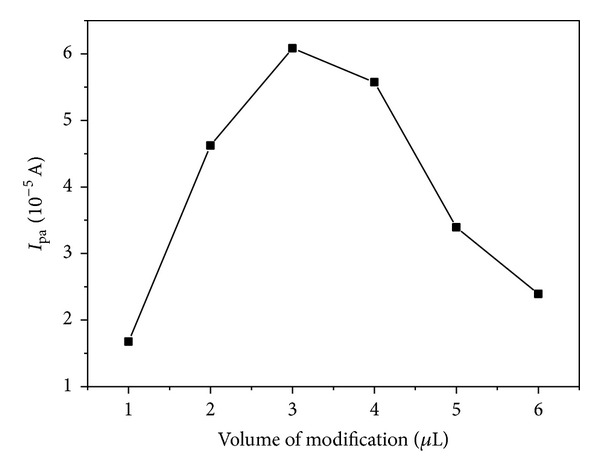
The plots of the anodic peak currents against the volume of modification.

Figure 8 depicts the influence of the immobilized amount of PDDA-G dispersion (1 mg mL^−1^) on the oxidation peak current of FA. The oxidation peak current increased gradually with the volume of PDDA-G dispersion increasing till 3 *μ*L and then decreased with further increasing the amount of PDDA-G, suggesting that if the modified film is too thick, it is unbeneficial for FA sensing. The amount of PDDA increased simultaneously with the amount of graphene. The oxidation peak current would decrease when the promotion effect on electron transfer caused by graphene was weaker than the block effect generated by nonconducting PDDA. Therefore, 3 *μ*L of 1 mg mL^−1^ PDDA-G dispersion was selected as the optimal immobilization volume.

### 3.7. Calibration Curve and Detection Limit

**Figure 9:**
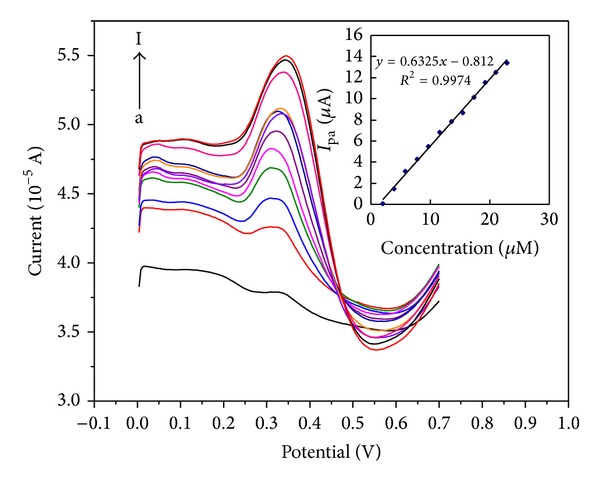
DPVs of FA with different concentrations (a: 0.0895; b: 4.29; c: 5.55; d: 7.92; e: 10.61; f: 11.56; g: 14.09; h: 14.88; i: 17.12; j: 19.94; k: 21.84; l: 52.92**** *μ*M) on PDDA-G/GCE in pH 3.0 acetate buffer solution. Conditions: scanned from 0 V to 0.7 V with amplitude of 0.05 V and pulse width of 0.05** **s. Inset: the calibration curve between the anodic peak currents with the concentration of FA.

DPV, as a kind of more sensitive techniques compared with CV, was chosen for the quantitative analysis of FA in real samples. As shown in Figure 9, under optimal conditions, the oxidation peak current was proportional to FA concentration at 8.95 × 10^−8^ M to 5.29 × 10^−5^ M, and the linear regression equation can be expressed as *I*_*p*_ (*μ*A) = 0.6325C (*μ*M)−0.812, *R* ^2^ = 0.9974. The detection limit (S/*N* = 3) was estimated at approximately 4.42 × 10^−8^ M. This lower detection limit can be ascribed to the electrocatalytic activity and adsorption ability of PDDA-G to FA.

### 3.8. Reproducibility, Stability, and Selectivity

The reproducibility of the PDDA-G/GCE was evaluated in terms of relative standard deviation (RSD) by measuring the FA (5.86 × 10^−6^ M) response to six modified electrodes. Three repetitive calibration experiments were carried out with each electrode. The average peak current was 2.849 *μ*A, and the RSD was 4.5%, revealing that this method had good reproducibility. The long-term operational stability of PDDA-G/GCE was examined by measuring the electrode response with 5.86 × 10^−6^ M FA at an interval of 7 d. During intervals of measurement, the electrode was stored at 4°C in a refrigerator. The current response decreased to 96.67% after 7 d, whereas 95.36% of the original response was retained after 14 d.

Various ions and molecules were studied on the basis of their interferences in the determination of FA by using PDDA-G/GCE. The major potential interferences are some cations found in the real samples. The interference effects of glucose, uric acid, ascorbic acid, citric acid, L-glutamic acid, L-glycine, phthalate, and ligustilide were also examined. In this study, 10 mL of the acidic buffer containing 5.86 × 10^−6^ M FA and different quantities of possible interferences were prepared to record the differential pulse voltammograms (DPVs) of these solutions. The results are summarized in Table 1, showing that the performance of the developed sensor was not significantly affected by the presence of various interferences. The results of reproducibility, stability, and interference tests indicated that PDDA-G/GCE can be applied to detect FA in real samples. Ascorbic acid with high concentration interfered with the detection of FA, probably because this substance increased the reduction products of FA in electrolytes. A possible reaction mechanism involved the participation of oxidation products of FA in electrochemical reduction and chemical reduction simultaneously, causing the increase of oxidation peak current accompanied by the decrease of reduction peak current.

### 3.9. Analytical Application

To validate the present method, PDDA-G/GCE was applied to the determination of FA in* Angelica sinensis* and urine samples. The results were listed in Table 2, respectively. For verifying the feasibility and reliability of the proposed method, high-performance liquid chromatography was used to detect the FA content in* Angelica sinensis*, and the results were consistent with those of our method. These results confirmed that PDDA-G/GCE was suitable for accurate, rapid, and sensitive determination of FA in pharmaceutical samples and clinical analysis.

According to the literature, several analytical methods have been employed for the detection of FA, from liquid chromatography to electrochemical techniques. By comparing the common analytical parameters (Table 3), such as the linear range and the limit of detection (LOD), it was observed that the present method showed a better performance for the detection of FA than most previously reported systems.

## 4. Conclusions

In this study, an electrochemical sensor for FA based on the PDDA-G/GCE was successfully fabricated, and the performance of this sensor was dramatically improved owing to the excellent electrical conductivity, strong adsorptive ability, and large effective surface area of PDDA-G. The sensor was applied to detect FA in* Angelica sinensis* and urine samples with satisfactory recoveries from 96.09% to 102.39%. Therefore, this study will provide a novel and promising platform for broadening the application of graphene in the field of pharmaceutical analysis.

## Figures and Tables

**Table 1:** Interference of different species in the determination of FA at PDDA-G/GCE.

Interference	Interference concentration/*μ*g mL^−1^	FA recovery/%
Glucose	36.37	102.23
Uric acid	13.52	99.87
Ascorbic acid	29.03	98.69
Citric acid	39.58	100.14
L-Glutamic acid	33.34	102.52
L-Glycine	16.08	96.57
Phthalate	15.74	104.71
Ligustilide	78.38	102.48

**Table 2:** Detection of FA in *Angelica sinensis* and in urine samples (*n* = 3).

Sample	Content/*μ*g mL^−1^	Detected/*μ*g	Spiked/*μ*g	Recovery/%	RSD/%
*A. sinensis *					
1	1.65	32.78	16.02	101.75	3.72
2	1.61	31.36	15.86	96.09	2.46
3	1.70	32.87	15.48	102.39	4.25
Urine					
1	0.55	5.54	5.58	99.28	0.37
2	1.15	11.45	11.68	98.03	1.37
3	1.72	17.19	16.89	101.78	4.72
4	3.29	32.91	33.24	99.01	4.96

**Table 3:** Comparison of different analytical methods for the detection of FA.

Linear range (*μ*g mL^−1^)	LOD (*μ*g mL^−1^)	Detection method	References
9.71–194.18	0.19	SWSV^a^	[14]
0.097–15.53	0.023	IPM^b^	[15]
0.18–582.54	0.06	DPV	[16]
0.04–2.14	7.77 × 10^−3^	CSV^c^	[17]
0.50–20.0	0.04	HPLC-DAD-ESI/MS^d^	[42]
0.05–5.0	0.01	CZE (amperometric)^e^	[43]
0.10–9.71	0.04	DPV	[32]
0.02–10.27	8.58 × 10^−3^	DPV	Present

^a^Square wave stripping voltammetry; ^b^irreversible biamperometry; ^c^cathodic stripping voltammetry; ^d^high-performance liquid chromatography coupled with diode array detection and electrospray ionization mass spectrometry; ^e^capillary zone electrophoresis.
